# A Critical Overview of Interleukin 32 in Leishmaniases

**DOI:** 10.3389/fimmu.2022.849340

**Published:** 2022-03-03

**Authors:** Fátima Ribeiro-Dias, Iara Barreto Neves Oliveira

**Affiliations:** Laboratório de Imunidade Natural, Instituto de Patologia Tropical e Saúde Pública, Universidade Federal de Goiás, Goiânia, Brazil

**Keywords:** IL-32, *Leishmania*, tegumentary leishmaniasis, visceral leishmaniasis, trained immunity, genetic, vitamin D

## Abstract

Interleukin-32 (IL-32) has several immune regulatory properties, which have driven its investigation in the context of various diseases. IL-32 expression is reported to be induced in the lesions of patients with American tegumentary leishmaniasis (ATL) by the New World *Leishmania* spp. that are responsible for causing ATL and visceral leishmaniasis (VL). IL-32 expression may elevate the inflammatory process through the induction of pro-inflammatory cytokines and also *via* mechanisms directed to kill the parasites. The genetic variants of IL-32 might be associated with the resistance or susceptibility to ATL, while different isoforms of IL-32 could be associated with distinct T helper lymphocyte profiles. IL-32 also determines the transcriptional profile in the bone marrow progenitor cells to mediate the trained immunity induced by β-glucan and BCG, thereby contributing to the resistance against *Leishmania*. IL-32γ is essential for the vitamin D-dependent microbicidal pathway for parasite control. In this context, the present review report briefly discusses the data retrieved from the studies conducted on IL-32 in leishmaniasis in humans and mice to highlight the current challenges to understanding the role of IL-32 in leishmaniasis.

## Introduction

Interleukin 32 (IL-32) (1), which was previously known as the Natural Killer (NK) cell transcript 4 (NK4), is a cytokine secreted by both immune and non-immune cells. It was previously recognized as a pro-inflammatory cytokine. However, the existence of different isoforms of IL-32 has revealed that besides its pro- or anti-inflammatory properties, IL-32 also possesses regulatory properties (2, 3). The role of IL-32 has been, so far, investigated in several inflammatory and infectious diseases (4–8), including different leishmaniases (9–15).

Leishmaniases refer to a group of diseases that are caused by the *Leishmania* protozoa, which comprises various species with different geographic distributions across the world. These different species may be associated with diverse clinical forms of leishmaniasis, the most frequently detected ones among which are cutaneous (CL) and mucosal (ML) leishmaniases, also referred to as tegumentary leishmaniasis, and visceral leishmaniasis (VL) (16). In leishmaniasis, the disease outcomes depend on several factors, including different mammal reservoirs, vectors, parasite species, and host status. In regard to the host status, the immune responses are responsible for parasite control and also immunopathogenesis. In this context, the role of IL-32 was investigated in infection with *Leishmania* sp., which indicated IL-32 as a crucial player in the immune responses against this parasite.

In the present review, the studies on IL-32 in leishmaniases are discussed briefly, with a particular focus on the current challenges encountered in this field, including the high number of IL-32 isoforms with different properties and interactions, the lack of a known IL-32 receptor to date, and the difficulties associated with murine models.

## Biology of IL-32

IL-32 is considered a pro-inflammatory cytokine, despite there being no sequence homology to any of the other cytokine families (1). While IL-32 is mainly reported as a human/primate-specific gene (17), its expression has been detected in other mammals as well, although not in rodents (1, 18). The biological activity of IL-32 is conserved across different species. Although rodents do not exhibit IL-32 expression, their cells are able to respond to IL-32 and subsequently produce several pro-inflammatory cytokines (1, 19).

IL-32 is predominantly intracellular, although it may also be secreted depending on the isoform, cell type, and context. While the exact localization of IL-32 inside a cell has not been determined so far, its co-localization with lysosomes (10), endoplasmic reticulum (20), Golgi apparatus (21), and mitochondria are reported (22). The *IL32* gene contains eight exons, and different isoforms are generated through alternative mRNA splicing. To date, IL-32*α*, IL-32*β*, IL-32*γ*, IL-32*δ*, IL-32*θ*, IL-32*ϵ*, IL-32*ζ*, IL-32*η*, and IL-32s isoforms have been identified. IL-32*γ* is considered the most potent isoform, capable of inducing higher tumor necrosis factor (TNF-a) production compared to the other isoforms (23). The interaction between these isoforms results in the functional diversity of IL-32 (24). For instance, IL-32*δ* interacts with IL-32*β* and inhibits IL-10 induction through this isoform (25).

IL-32 is produced by immune cells (such as macrophages, monocytes (26), NK cells (27), and T lymphocytes) (28), as well as by non-immune cells (epithelial (29), endothelial (20, 30), mesenchymal stromal cells (31), and fibroblasts) (32). Certain tumor cell lines express IL-32 constitutively (33–35). Pro-inflammatory cytokines such as (TNF-*α*; 19), IL-12 (1), IL-18 (4), and IL-1*β* (36) induce IL-32 production. IL-32 is also expressed in viral (37–39), bacterial (40, 41), fungal (42), and protozoan infections (9). Pathogen-associated molecular patterns, such as lipopolysaccharide (LPS), muramyl dipeptide (MDP), RNA analog Poly (I:C), and oxidative stress, also induce IL-32 (21, 26, 43, 44). In turn, IL-32 induces TNF-*α*, macrophage inflammatory protein 2, IL-8, and IL-1*β*, *via* NF-*κ*B, AP-1, and p38-MAPK signaling pathways, in monocytes/macrophages (1, 36), synovial fibroblasts (4, 45), and T cells (1, 19, 46). In epithelial cells, IL-32*γ* acts synergistically with the NOD1/2 receptors and induces IL-1*β* secretion (47). In fibroblast-like synoviocytes, IL-32*γ* upregulates TLR2 and NOD2, thereby inducing IL-1*β* in response to the cell wall components of *Streptococcus pyogenes* (48). Moreover, IL-32*γ* promotes the differentiation of monocytes into macrophages or dendritic cells (DC) (49, 50).

Proteinase 3 (PR3)–proteinase-activated receptor 2 (PR2) axis is the main IL-32 receptor candidate. PR3 was reportedly activated by IL-32*γ*, leading to the activation of the G protein-coupled receptor PR2, which then induced a cytokine response *via* Ras-Raf and TRIF (51, 52). PR3 exhibits affinity to IL-32*α* (51) and IL-32*γ* (53) and is expressed mainly in neutrophils. The tripeptide motif Arg-Gly-Asp (RGD) present in the IL-32 isoforms occurs in different accessibility forms (54) that allow interaction with the integrin present on the cell surface, adhesion regulation, migration (55), apoptosis, and angiogenesis (56). IL-32*α*, IL-32*β*, and [to a lesser extent] IL-32*γ* bind to the extracellular domain of integrin *α*V*β*3 (54). The binding between RGD and integrin activates the intracellular kinases, such as focal adhesion kinase (FAK) (57), and may activate the *β*3-p38MAPK pathway (32). IL-32 also interacts directly with the focal adhesion protein paxillin (54).

Each IL-32 isoform may interact with a specific protein kinase C (PKC) to modulate gene expression. IL-32*α* interacts with PKCϵ and STAT3 (58). IL-32*β* interacts with PKC*ϵ* and C/EBP*α*, leading to IL-10 upregulation (59). IL-32*β* also binds to the proto-oncogene Src in breast cancer cells to induce glycolysis (27). IL-32*θ* interacts with PKC*δ*, decreasing CCL5 production *via* STAT3 phosphorylation (60). IL-32*θ* inhibits the PKC-*δ*–mediated pathways responsible for TNF-*α* and IL-1*β* production (1, 61).

IL-32*γ* appeared to protect against *in vivo Mycobacterium tuberculosis* (MTB) infection in humans and IL-32 transgenic mice (6, 41). However, this effect declined at the later stages of infection, when the mRNA of IL-32*γ* was spliced into IL-32*β* mRNA, which increased the levels of IL-10-expressing macrophages or DCs (41). For its protective effects, besides apoptosis induction (62), IL-32*γ* also induces the expression of 25-hydroxyvitamin D3 1-alpha-hydroxylase (CYP27B1), which converts inactive vitamin D (25D) into bioactive 1,25-dihydroxy vitamin D3, which then binds to VDR (vitamin D receptor) and increases the production of antimicrobial peptides cathelicidin and *β*-defensin (6). IL-32*γ* is reported to protect against other mycobacterial infections as well (26, 63).

In human *IL-32* transgenic murine models, IL32*β* reportedly increased the inflammation and worsened sepsis (17), besides inducing neuroinflammation in the brain (64). The pro-inflammatory activity of IL32*β* was also observed in arthritis and colitis mouse models (19). In contrast, IL-32*β* exhibited anti-inflammatory effects by reducing arthritis (65) and tumor growth in IL32*β*-transgenic mice (64) or *in vivo* arthritis model (45) as well as colitis (66) and protection against tuberculosis in IL-32*γ*-transgenic mouse models (41).

In HIV infection, IL-32*γ* induced viral production in latently-infected CD4^+^ T cells (67). The *IL32* single-nucleotide polymorphism (SNP) rs4349147 has been associated with HIV susceptibility (68). In a study, the G allele-bearing cells that exhibited a shift to IL-32 isoforms other than IL-32*α*, such as IL-32*γ* or IL-32*β*, became cells prone to HIV infection (7). In hepatitis virus (HCV and HBV) infections, IL-32 appears to contribute to inflammation and fibrosis by inducing pro-inflammatory cytokines (69), apoptosis (70), B7-H6 expression on hepatocytes (71), and interferon IFN-Λ1 (72). Intracellular IL-32 inhibits HBV replication and downregulates the transcription factors essential for HBV *via* the ERK1/2 pathway (73).

## IL-32 in Different Leishmaniases

### American Tegumentary Leishmaniasis (ATL)

IL-32 is highly expressed in lesions in both CL and ML patients (9). The IL-32 protein and IL-32γ mRNA detected in mucosal lesions were reportedly associated with TNF-α expression, indicating a role of IL-32 in the immunopathogenesis of ATL. IL-32 has been detected in the mononuclear cells of the inflammatory infiltrate and also in non-immune cells such as epithelial and endothelial cells. Amastigote forms of *L*. *braziliensis* induced IL-32γ mRNA in PBMCs from healthy individuals within 24 h of incubation (9). Therefore, while IL-32γ is produced immediately upon the initial interactions of immune cells with parasites, it may also be detected during chronic inflammation. In addition, IL-32 was highly detected in skin lesions of patients infected with *L. amazonensis* (12).

**Figure 1:**
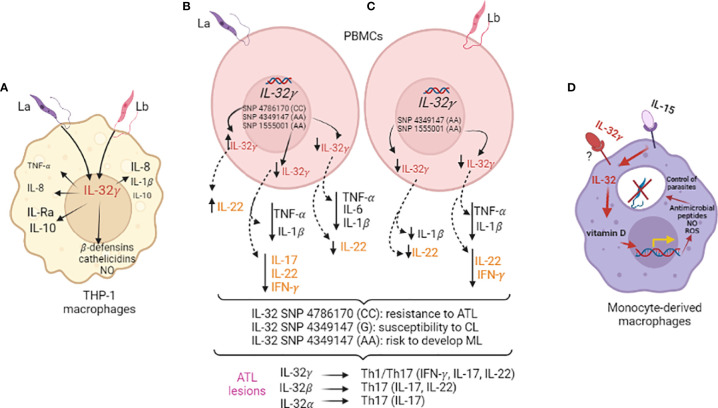
Interleukin-32 in *Leishmania* infections. **(A)** Human macrophages derived from the monocyte cell line THP-1 were infected with *L. amazonensis* (La) or *L. braziliensis* (Lb) (MOI 5:1) after IL-32γ silencing (siRNA) or overexpression (plasmid) for evaluation of cytokine and antimicrobial molecules. **(B, C)** PBMCs from healthy individuals genotyped for three *IL32* variants were exposed to La **(B)** or Lb **(C)** promastigote lysates for cytokine evaluation (innate - TNF-α, IL-1β, IL-6/24 h; acquired immunity: IL-22, IL-17, IFN-γ/seven days) and association with ATL and its clinical outcome; expression of cytokines and different IL-32 isoforms were evaluated in lesions of ATL patients and positive correlations were obtained. **(D)** Human monocyte-derived macrophages were infected with Lb after priming with recombinant cytokines - IL-15 (inducer of IL-32) and/or IL-32γ to evaluate vitamin D-dependent microbicidal pathway and NO and ROS production.

Upon IL-32γ silencing or overexpression in the human monocytic THP-1 cell line, early expression of IL-32γ mRNA was confirmed for *L*. *braziliensis* and *L*. *amazonensis*, and it was dependent on TNF-α. Moreover, the expressions of TNF-α mRNA and IL-8 mRNA and protein induced by each *Leishmania* sp. were dependent on IL-32γ. However, TNF-α was produced at similar levels upon exposure to *L. amazonensis* or *L. braziliensis* in an IL-32γ-independent manner. Only *L. braziliensis* could induce IL-1β production independent of IL-32γ. The IL-1 receptor antagonist (IL-1Ra) mRNA and protein levels and IL-10 mRNA levels were higher after exposure to *L. amazonensis* compared to *L. braziliensis*, and only *L. amazonensis*-induced IL-1Ra was affected by IL-32γ expression (10). These findings suggested that the effect of IL-32γ expression on cytokine production differs with the *Leishmania* species. In the absence of IL-32γ, the infection index increased, which was attributed to the decreased levels of iNOS/NO (nitric oxide) and antimicrobial peptides (β-defensin-2 and cathelicidin). Reactive oxygen intermediates (ROS) and antimicrobial peptides are reported to kill *Leishmania* (74, 75). Accordingly, IL-32γ overexpression led to better parasite control together with increased production of microbicidal molecules (**Figure 1A**) (10). Therefore, IL-32γ expression is crucial for parasite control against both the *Leishmania* species.

Dos Santos et al. (14) demonstrated that both *L. amazonensis* and *L. braziliensis* could induce IL-32γ mRNA in the PBMCs from healthy individuals within 24 h of incubation, while IL-32β and IL-32α mRNA were detected only after seven days. This suggested that isoforms other than IL-32γ could be produced during *in vivo* infection, which could differentially modulate the immune responses. The search for the innate receptors responsible for IL-32 induction revealed that toll-like receptor 4 (TLR4), NOD2, and Dectin-1 recognized *Leishmania* molecules for IL-32 induction (14). Lipophosphoglycan (LPG) from *Leishmania* activates TLR4 and NOD-like receptors (NLRP3) to increase cytokine production (76–78). Therefore, LPG is a suitable candidate parasite-derived molecule for inducing IL-32 production during *Leishmania* infection.

The major challenge in the study of the mechanisms and biological activities of IL-32 in infectious diseases is the lack of a known receptor for IL-32. Strategies other than those aiming at the blocking/inhibition of cytokine receptor/signaling are required to investigate the role of IL-32 in leishmaniases. In this context, a genomic functional study allows identifying the genetic variants of IL-32 (79) capable of regulating its production and influencing the development and disease outcome in tegumentary leishmaniasis. Three *IL32* variants already evaluated in other diseases (7, 80–82) were investigated in ATL. A Brazilian cohort of ATL patients and healthy individuals were evaluated for *IL32* SNP rs4786370 (promoter region), which is associated with protection against ATL. When PBMCs from healthy individuals (200FG cohort; 79; http://www.humanfunctionalgenomics.org/site/) were exposed to *L. amazonensis* lysate, the CC genotype of *IL32* rs478670 exhibited increased IL-32γ mRNA expression. Nonetheless, the production of innate (IL-1β, TNF-α, IL-6) or acquired (IFN γ, IL-17) immunity cytokines remained unaltered. However, IL-22 levels increased in the CC genotype individuals (14).

While intronic *IL32* SNP rs1555001 and enhancer *IL32* SNP rs4349147 were not associated with ATL susceptibility or resistance, a decrease was observed in IL-32γ mRNA, TNF-α, IL-1β, IL-22, and IFN-γ expressions in *L. amazonensis*-or *L. braziliensis*-exposed PBMCs with the AA genotype of rs1555001 SNP. Moreover, *L. amazonensis* exposure led to decreased IL-17 induction. In the AA genotype of *IL32* SNP rs4349147, exposure to both *Leishmania* species led to decreased IL-32γ, IL-1β, and IL-22 levels, while TNF-α and IL-6 levels decreased only upon *L. amazonenis* exposure. The SNPs rs4786370 and rs1555001 were not associated with clinical outcomes; the *IL32* SNP rs4349147 allele G was present at high frequency in CL patients, while allele A was overrepresented in ML patients (14). These findings, together with the observations that these cytokines are highly expressed in ATL lesions and IL-32 isoforms are associated with innate and acquired cytokines, suggest that increased IL-32γ and IL-22 levels protect against ATL, while decreased levels of these cytokines represent a risk for ML. In murine models, IL-22 is considered irrelevant to parasite control (83, 84), although it does facilitate healing of the lesions, thereby protecting against tissue damage (84). The three *IL32* SNPs evaluated in Dos Santos et al. (14) affected the expression of IL-22, which is strongly associated with IL-32γ in ATL lesions. Notably, while IL-32γ was associated with IFN-γ and IL-17 (Th1/Th17 profile), IL-32β was associated with IL-22 and IL-17, and IL-32α with IL-17 (Th17 profile) (14) (**Figures 1B, C**). Therefore, the isoforms could be determinant to the acquired immune responses against *Leishmania*.

The role of IL-32 in the clinical outcome of diseases has been evaluated in human IL-32 transgenic mice. Choi et al. (66) used the mouse strain C57BL/6 to develop IL-32γ transgenic mice (IL-32γTg), in which the chicken β-actin promoter drives IL-32γ expression in all tissues. In this mouse model, IL-32γ promoted parasite control and lesion healing after infection with *L. braziliensis*, and was associated with the increased production of Th1 cytokines (12). While IL-32γ did not contribute to the healing of lesions caused by *L. amazonensis*, it decreased parasite dissemination from the footpad to the liver or spleen (12). This mouse model was also used to evaluate the role of IL-32 in trained immunity as an alternative mechanism to enhance protection against *Leishmania*. In the study, β-glucan was first used for training the human monocytes (85), which induced IL-32γ mRNA expression and IL-32 production while also enhancing the control of *L. braziliensis*. These results were mechanistically explained by an increase in the expression of antimicrobial peptides cathelicidin and β-defensin-2. The stratification of healthy individuals according to their *IL32* SNP rs4786370 genotype (200FG cohort; 79) revealed that the CC genotype expressed higher levels of IL-32γ, IL-1β, IL-6, and TNF-α in β-glucan-trained macrophages compared to the TT genotype. The presence of this *IL32* variant was also associated with a decreased infection index. These findings, together with the genomic functional data, suggested that β-glucan induces IL-32 and IL-1β, which then mediate trained immunity and enhance protection against *L. braziliensis* infection (85).

In IL-32γTg mice, β-glucan training increased resistance against *L. braziliensis* infection, which was mechanistically explained by an increased expression of the genes associated with cell cycle, myeloid lineages, and regulatory enzymes of the glycolytic pathway in bone marrow cells. Similar results were reported for human BCG-vaccinated volunteers genotyped for *IL32* SNP rs4786370, in which the bone marrow myeloid progenitor cells with CC genotype presented an association of IL-32 expression with increased metabolic gene expression, besides inflammation (86). BCG reportedly induces IL-32 (40, 87), while BCG-trained monocytes exhibit enhanced capacity to kill *L. braziliensis*, *L. amazonensis*, and *L. infantum* through increased ROS production (86). Moreover, IL-32γTg mice trained with BCG exhibit resistance to *L. braziliensis* and *L. infantum*, and control of *L. amazonensis* dissemination. This was associated with increased inflammation (87). Data indicate that IL-32 serves as a determinant of gene expression profile at the level of bone marrow progenitors by mediating the trained immunity induced by β-glucan or BCG and conferring protection against leishmaniasis. Dos Santos et al. reported a review on BCG in leishmaniasis (88).

One of the microbicidal pathways driven by IL-32γ is the vitamin D-dependent production of antimicrobial peptides. *L. braziliensis* infection in human monocyte-derived macrophages was reportedly best controlled when the culture medium contained IL-32γ and a sufficient amount of vitamin D (**Figure 1D**) (15). However, this pathway appears important in the control of various microorganisms in human macrophages as the IL-32–vitamin D axis is also crucial for controlling the growth of *Paracoccidioides brasiliensis*, a fungus that causes paracoccidioidomycosis (42).

Although IL-32γTg mice have been useful in studying the role of IL-32 in leishmaniases, the differences between humans and mice render these unsuitable as an optimal model. For instance, the vitamin D pathway in mice lacks the induction of β-defensin-2 and cathelicidin (89), consequently limiting the use of this IL-32-dependent pathway in IL-32γTg mouse in analyses. Moreover, mice and humans differ in the NO levels generated after *Leishmania* infection and their relevance in parasite control (15, 90, 91). Therefore, the effects of IL-32 on microbicidal pathways could differ between humans and mice.

### Visceral Leishmaniasis (VL)

*Leishmania infantum* promastigotes may induce high levels of IL-32γ expression and IL-32 production and low levels of IL-32β in human PBMCs. In IL-32γTg mice, IL-32γ expression was reportedly increased in the liver and spleen, which ultimately reduced the parasite burden and increased granuloma formation in the liver, compared to wild-type mice. The protection was associated with Th1 and Th17 cells, which produced cytokines that contributed to NO production (13). IL-32γ also enhanced the protective role of neutrophils in VL. IL-32γ increased the number of neutrophils in mouse spleen and liver after *L. infantum* infection *via* IL-17-dependent cell recruitment. Recombinant IL-32γ increased ROS production in both mouse and human neutrophils (92). Together, data suggest a crucial role of the IL-32γ–Th17–neutrophil axis in the control of experimental VL. As stated above, IL-32 appears to mediate the BCG-trained immunity, which confers protection against *L. infantum* to human monocytes/macrophages and IL-32γTg mice (87). However, data on the role of IL-32 in the course of human VL deserve investigation.

## Conclusion

Studies have demonstrated that IL-32 plays a crucial role in leishmaniases. However, studies attempting to unravel the role of IL-32 in leishmaniases and other diseases encounter certain major challenges, including the high number of IL-32 isoforms, each with distinct biological properties, lack of a recognized IL-32 receptor, the large sample size required for genetic studies, few individuals with *IL32* variants available for functional studies, and the differences in the IL-32γ-mediated microbicidal pathways between humans and mice. Future studies should investigate the presence of other IL-32 isoforms, *in vivo*, during microbial infection and attempt to identify IL-32 receptors to unravel the mechanisms through which IL-32 modulates immune responses during infection with different *Leishmania* species.

## Author Contributions

FR-D was responsible for designing, organizing and, revising the manuscript. Both authors contributed to the literature review, analyzes the studies, writing of the manuscript, and approved the submission.

## Funding

Coordenação de Aperfeiçoamento de Pessoal de Nível Superior - Brazil (CAPES) – Finance Code 001, by FAPEG - grant n. 465771/2014-9 - INCT/IPH – National Institute of Science and Technology for strategies in host-pathogen interaction, Brazil. FAPEG - PRONEM grant n. 2017 10267000516.

## Conflict of Interest

The authors declare that the research was conducted in the absence of any commercial or financial relationships that could be construed as a potential conflict of interest.

## Publisher’s Note

All claims expressed in this article are solely those of the authors and do not necessarily represent those of their affiliated organizations, or those of the publisher, the editors and the reviewers. Any product that may be evaluated in this article, or claim that may be made by its manufacturer, is not guaranteed or endorsed by the publisher.
